# Cerebrospinal Fluid Autoantibody Profiles in Rheumatologic Disorders Affecting the Central Nervous System: A Systematic Review

**DOI:** 10.7759/cureus.98318

**Published:** 2025-12-02

**Authors:** Taher Mohammed, Sharmila Venkatachalapathi, Kiranjot Kaur, Mashal Mumtaz, Sabahat Iftikhar, Mahvish Nishat, Nada Rashid, Ensaf Ibrahim Hassan Ibrahim, Abdullah Khan

**Affiliations:** 1 Medical Education, StepExcel Boards Academy, Skokie, USA; 2 Internal Medicine, Government Periyar Hospital, Mayiladuthurai, IND; 3 Internal Medicine, Vadamalayan Hospitals Pvt. Ltd., Dindigul, IND; 4 Medicine, US Navy, United States Military, Great Lakes, USA; 5 Clinical Research, Arizona State University, Tempa, USA; 6 Medicine, Shri B. M. Patil Medical College, Bijapur, IND; 7 Internal Medicine, University College of Medicine and Dentistry, University of Lahore (UoL), Lahore, PAK; 8 Medicine, StepExcel Boards Academy, Skokie, USA; 9 General Physician, Shadan Institute of Medical Science, London, GBR; 10 Internal Medicine, National Health Service (NHS), Wakefield, GBR; 11 Rheumatology, Royal Derby Hospital, Derby, GBR; 12 Internal Medicine, Sheikh Zayed Hospital, Rahim Yar Khan, PAK

**Keywords:** autoantibodies, biomarkers, cns, csf, nmosd, npsle, rheumatologic disorders, sle

## Abstract

Rheumatologic disorders affecting the central nervous system (CNS) can cause significant neurological and psychiatric morbidity. Early detection of CNS involvement is essential to prevent irreversible damage. This study aims to systematically review cerebrospinal fluid (CSF) autoantibody profiles in rheumatologic disorders and evaluate their diagnostic, prognostic, and clinical relevance. A systematic review of PubMed, Embase, Scopus, and Cochrane Library was conducted up to October 2025. Studies reporting CSF autoantibodies in systemic lupus erythematosus (SLE), primary Sjögren’s syndrome (pSS), neuromyelitis optica spectrum disorder (NMOSD), and rheumatoid arthritis (RA) with CNS involvement were included. Data on antibody type, clinical correlations, and prognostic significance were extracted and synthesized. Ten studies met the inclusion criteria. Anti-NR2 and anti-ribosomal P antibodies were associated with neuropsychiatric manifestations in SLE. Anti-SSA (Ro) indicated CNS involvement in pSS. CSF aquaporin-4 IgG correlated with active disease in NMOSD, while anti-cyclic citrullinated peptide antibodies were detected in RA-related meningitis. CSF autoantibodies reflected intrathecal immune activity and offered higher diagnostic specificity than serum measurements. CSF autoantibody profiling provides clinically relevant biomarkers for CNS involvement in rheumatologic disorders. Standardized, longitudinal studies are needed to develop predictive panels and guide personalized immunotherapy.

## Introduction and background

Rheumatologic disorders affecting the central nervous system (CNS) are a heterogeneous group of autoimmune diseases that can lead to significant neurological and psychiatric morbidity [1]. These disorders, including systemic lupus erythematosus (SLE), primary Sjögren’s syndrome (pSS), neuromyelitis optica spectrum disorder (NMOSD), and rheumatoid arthritis (RA) with CNS involvement, often present with complex clinical phenotypes. Manifestations may range from subtle cognitive impairments and mood disturbances to severe neuropsychiatric syndromes, seizures, and demyelinating events. Early recognition is crucial as delayed diagnosis can result in permanent neurological deficits and increased mortality [2]. The overlapping symptoms with non-autoimmune neurological disorders, such as multiple sclerosis, infections, or vascular events, often complicate clinical evaluation. Therefore, identifying reliable biomarkers is essential to enhance diagnostic accuracy and improve patient outcomes.

Cerebrospinal fluid (CSF) provides a direct window into CNS pathology, reflecting intrathecal immune activity [3]. Autoantibodies detected in the CSF have been increasingly recognized as valuable indicators of CNS involvement in rheumatologic disorders [4]. In SLE, for instance, anti-NR2 (a subunit of N-methyl-D-aspartate receptor) and anti-ribosomal P antibodies have been associated with neuropsychiatric manifestations. Similarly, anti-Sjogren’s syndrome-related antigen (anti-SSA) A (Ro) antibodies in pSS and Aquaporin-4 Immunoglobulin G (AQP4-IgG) in NMOSD are linked with CNS pathology and can help distinguish these disorders from other neurological conditions. These autoantibodies not only aid in diagnosis but may also correlate with disease severity, prognosis, and therapeutic response, making them potential tools for personalized patient management.

Despite growing research, the literature on CSF autoantibody profiles across various rheumatologic CNS disorders remains fragmented and disease-specific [5]. Most studies focus on single disorders such as SLE or NMOSD, limiting the ability to compare antibody patterns across conditions [6]. Additionally, variations in method, sample size, and autoantibody detection techniques contribute to inconsistencies in reported findings. Consequently, clinicians face challenges in interpreting CSF autoantibody results in a broader context. A comprehensive synthesis of the available evidence is necessary to identify common and disease-specific biomarkers and to evaluate their clinical utility for diagnosis, prognosis, and therapeutic guidance.

This systematic review aims to provide an updated and integrated overview of CSF autoantibody profiles in rheumatologic disorders affecting the CNS. Specifically, it seeks to summarize the types of autoantibodies reported, their association with neurological and psychiatric manifestations, and their potential prognostic significance. By analyzing data across multiple rheumatologic conditions, the review intends to highlight patterns, gaps in the literature, and areas for future research. The ultimate goal is to inform clinical practice and support the development of evidence-based diagnostic and management strategies for patients with CNS involvement in rheumatologic diseases. 

## Review

Materials and methods

Search Strategy

A comprehensive systematic literature search was conducted in PubMed, Embase, Scopus, and the Cochrane Library from database inception to October 2025 to identify studies reporting CSF autoantibodies in rheumatologic disorders affecting the central nervous system (CNS). The search combined MeSH terms and keywords, including “cerebrospinal fluid” OR “CSF,” “autoantibodies,” “rheumatologic disorders” OR “autoimmune rheumatic diseases,” “central nervous system” OR “CNS involvement,” and specific disease terms such as “systemic lupus erythematosus” OR “SLE,” “primary Sjögren’s syndrome” OR “pSS,” “neuromyelitis optica spectrum disorder” OR “NMOSD,” and “rheumatoid arthritis” OR “RA.” Boolean operators (AND, OR) were applied to combine terms, and filters were used to restrict results to human studies published in English. No restrictions were applied based on study design to include observational studies, case series, and cohorts reporting relevant CSF autoantibody data. Additionally, the reference lists of all eligible articles were manually screened to identify further studies. The study selection process followed Preferred Reporting Items for Systematic Reviews and Meta-Analyses (PRISMA) 2020 guidelines, ensuring transparent reporting of identification, screening, eligibility assessment, and inclusion of studies for qualitative synthesis [7].

Eligibility Criteria

The inclusion criteria for this systematic review were defined using the PICO framework [8]. The Population (P) comprised patients diagnosed with rheumatologic disorders affecting the CNS, specifically SLE, pSS, NMOSD, and rheumatoid arthritis (RA) with CNS involvement. The Intervention/Exposure (I) was the assessment of CSF autoantibodies, including disease-specific antibodies such as anti-NR2, anti-ribosomal P, anti-SSA (Ro), AQP4-IgG, and anti-CCP. The Comparator (C) included either patients with the same rheumatologic disorder without CNS involvement or non-autoimmune controls, when reported. The Outcomes (O) were the presence, levels, and clinical correlations of CSF autoantibodies with neurological or psychiatric manifestations, disease activity, and prognosis. Eligible studies were human studies published in English, including observational cohorts, prospective or retrospective clinical studies, and case series reporting relevant CSF autoantibody data. Studies were excluded if they involved animal models, case reports, or consisted of conference abstracts, editorials, or review articles without original patient data. This approach ensured the inclusion of studies with sufficient methodological rigor and sample size to allow meaningful synthesis and statistical evaluation of CSF autoantibody associations across rheumatologic CNS disorders.

Study Selection

All retrieved records were imported into a reference manager, and duplicates were removed. Titles and abstracts were screened independently by two reviewers for relevance. Full-text articles were then assessed against the predefined eligibility criteria. Disagreements were resolved through discussion or consultation with a third reviewer.

Data Extraction

A standardized data extraction form was used to collect study characteristics, patient population, CSF autoantibody types, outcomes, and follow-up. Two reviewers independently extracted the data to ensure accuracy. Missing or unclear information was clarified by contacting the corresponding authors when possible. Extracted data were cross-checked and verified before synthesis.

Risk-of-Bias Assessment

The methodological quality of the included studies was assessed using the Newcastle-Ottawa Scale (NOS) for observational studies [9] and the JBI checklist for case series [10]. Two reviewers independently evaluated each study for potential bias in selection, measurement, and outcome reporting. Discrepancies were resolved by consensus. Studies were categorized as low, moderate, or high risk of bias.

Data Synthesis

A narrative synthesis was performed due to heterogeneity in study design, CSF autoantibody assays, and outcomes. Autoantibody prevalence, clinical associations, and prognostic significance were summarized across studies. When sufficient data were available, quantitative pooling or descriptive statistics were presented. Findings were interpreted in the context of disease-specific and cross-disease patterns.

Registration 

This systematic review was not registered with PROSPERO (International Prospective Register of Systematic Reviews)

Results

**Figure 1:**
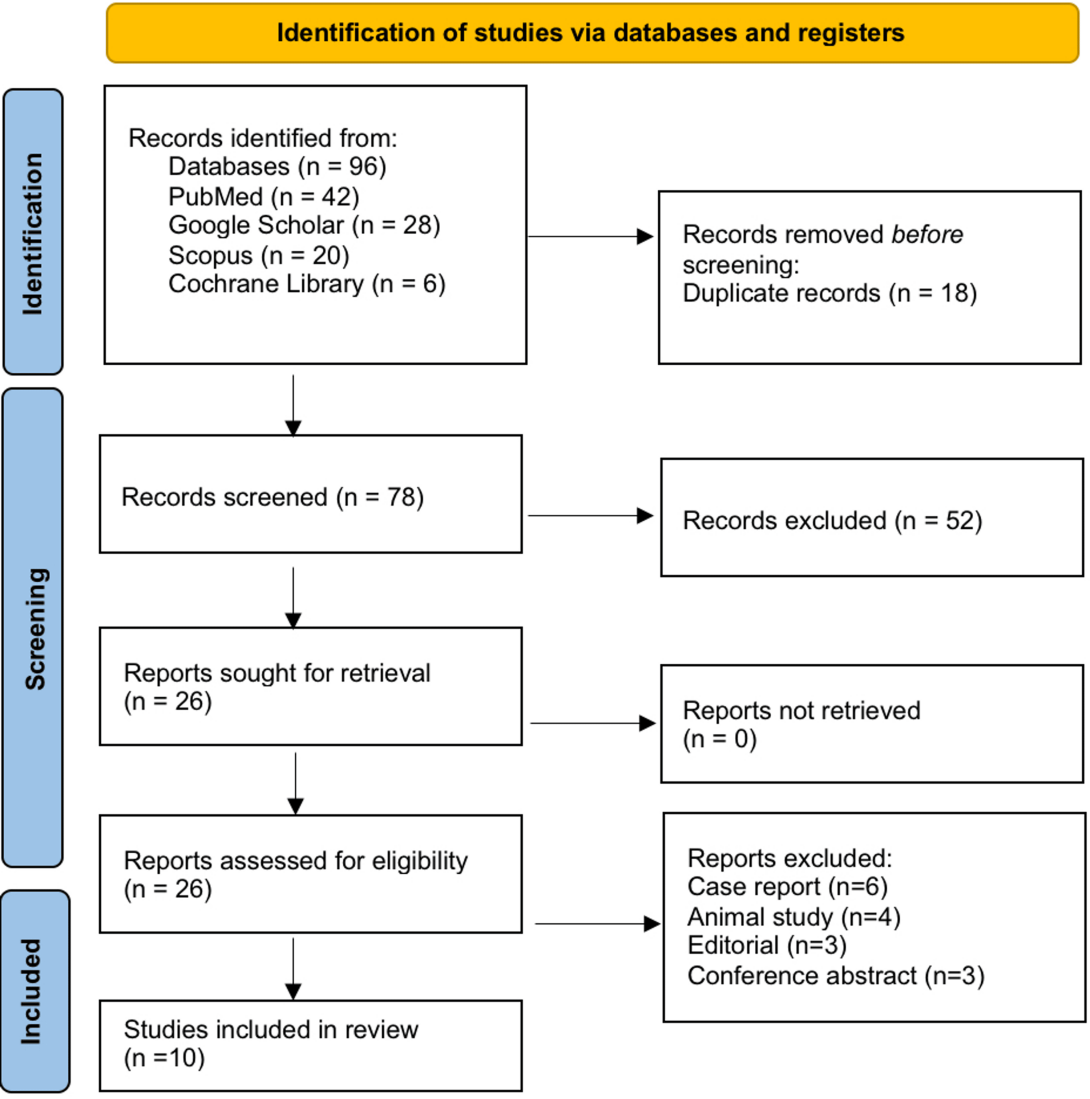
PRISMA 2020 flow diagram. PRISMA, Preferred Reporting Items for Systematic Reviews and Meta-Analyses

Figure 1 shows that a total of 96 records were identified through the systematic search of databases, including PubMed (*n* = 42), Embase (*n* = 28), Scopus (*n* = 20), and the Cochrane Library (*n* = 6). After removing 18 duplicate records, 78 unique records were screened based on titles and abstracts, of which 52 were excluded for being irrelevant to CSF autoantibodies in rheumatologic CNS disorders. Full-text versions of the remaining 26 articles were sought for retrieval, all of which were available and assessed for eligibility. Of these, 16 studies were excluded for the following reasons: case reports with fewer than three patients (*n* = 6), animal studies (*n* = 4), editorials (*n* = 3), and conference abstracts (*n* = 3). Ultimately, 10 studies met the predefined inclusion criteria and were included in the qualitative synthesis. The study selection process was performed according to PRISMA 2020 guidelines to ensure transparent and reproducible reporting.

Characteristics of the Selected Studies

Table 1 presents the characteristics of the studies included in this systematic review. Fragoso-Loyo et al. evaluated 47 patients with SLE and neuropsychiatric manifestations and found that CSF anti-NR2 antibodies were strongly associated with central neuropsychiatric SLE, reflecting the clinical course [11]. Yoshio et al. reported that CSF anti-NR2 levels correlated with neuropsychiatric manifestations in adults with NPSLE compared to non-NPSLE or neurological controls [12], while Hirohata et al. demonstrated that CSF anti-ribosomal P antibodies were linked to diffuse psychiatric syndromes in 71 patients with SLE [13]. Arinuma et al. confirmed a significant association between CSF anti-NR2 and neuropsychiatric manifestations in patients with SLE with or without NP features [14]. Hu et al. used proteome microarray analysis in 29 CSF samples to identify 137 novel autoantibodies, highlighting intrathecal autoimmunity in NPSLE [15]. Duarte-García et al. found that higher CSF anticardiolipin IgG and CCL2 levels correlated with cognitive dysfunction in SLE [16], whereas Mégevand et al. reported intrathecal synthesis of CSF anti-SSA antibodies in pSS patients with CNS involvement [17]. In NMOSD, Jarius et al. and Sato et al. demonstrated that CSF AQP4-IgG was detectable during attacks and correlated with pleocytosis and IL-6, serving as a marker of active disease [18,19]. Caputi et al. described positive CSF anti-CCP indices in two RA patients with rheumatoid meningitis, which declined after therapy [20]. These studies collectively highlight disease-specific CSF autoantibody profiles and their potential diagnostic and prognostic relevance across rheumatologic CNS disorders.

**Table 1:** Characteristics of the selected studies. CNS, central nervous system; CSF, cerebrospinal fluid; SLE, systemic lupus erythematosus; NPSLE, neuropsychiatric systemic lupus erythematosus; pSS, primary Sjögren’s syndrome; NMOSD, neuromyelitis optica spectrum disorder; RA, rheumatoid arthritis; NP, neuropsychiatric; AQP4-IgG, Aquaporin-4 immunoglobulin G; anti-NR2, antibodies against N-methyl-D-aspartate receptor subunit 2; anti-SSA (Ro), anti-Sjögren’s syndrome A (Ro) antibodies; anti-CCP, anti-cyclic citrullinated peptide antibodies; IgG, immunoglobulin G; CCL2, chemokine (C-C motif) ligand 2; P0/P1/P2, ribosomal P protein subunits

Authors and year (link)	Population (P)	Exposure/condition (I)	Comparator (C)	Outcomes (O)	CSF autoantibody (measured)	Rheumatologic CNS disorder	Prognosis/key finding
Fragoso-Loyo et al., 2008 [11]	47 patients with SLE and neuropsychiatric manifestations + controls	Neuropsychiatric SLE (central/peripheral)	SLE without NP; non-autoimmune controls	Presence/levels of serum and CSF autoantibodies; six-month follow-up	Anti-NMDAR (anti-NR2), anti-dsDNA, anti-ribosomal P	Neuropsychiatric SLE	CSF anti-NR2 strongly associated with central NPSLE; antibodies paralleled clinical course
Yoshio et al., 2006 [12]	Adults with NPSLE	NPSLE	Non-NPSLE or neurological controls	Detection of anti-NR2 in CSF; correlation with NP manifestations	Anti-NR2 (NMDAR subunit)	NPSLE	CSF anti-NR2 associated with neuropsychiatric manifestations; intrathecal correlation
Hirohata et al., 2007 [13]	71 patients with SLE + 24 controls	NPSLE (diffuse psychiatric syndromes)	Non-inflammatory neurological controls	CSF anti-P detection and clinical correlation	Anti-ribosomal P (P0/P1/P2)	NPSLE (psychiatric presentations)	CSF anti-P associated with diffuse psychiatric syndromes; diagnostic value supported
Arinuma et al., 2008 [14]	Patients with SLE with/without NP manifestations	NPSLE	SLE without NP	CSF anti-NR2 levels and NP correlation	Anti-NR2	NPSLE	Significant association between CSF anti-NR2 and NP manifestations
Hu et al., 2015 [15]	29 CSF samples (12 NPSLE, 7 non-NPSLE, 10 controls)	NPSLE (various presentations)	Non-NPSLE SLE and non-SLE controls	Proteome microarray discovery of CSF autoantigens	Novel CSF autoantibodies (137 candidate antigens)	NPSLE	Broad panel identified; intrathecal autoimmunity evident
Duarte-García et al., 2018 [16]	SLE cohort with/without cognitive dysfunction	SLE with cognitive dysfunction	SLE without cognitive dysfunction	CSF and serum autoantibodies, cytokines, and correlation with cognition	Multiple (anticardiolipin IgG, CSF chemokines)	SLE (cognitive involvement)	Higher anticardiolipin IgG and CSF CCL2 are linked to dysfunction
Mégevand et al., 2007 [17]	Primary Sjögren’s syndrome (pSS) with CNS involvement	pSS with CNS involvement	pSS without CNS involvement/controls	CSF testing and intrathecal synthesis evidence	Anti-SSA (Ro) in CSF	Primary Sjögren’s syndrome (CNS disease)	CSF anti-SSA intrathecal synthesis; potential biomarker
Jarius et al., 2010 [18]	NMOSD/AQP4-IgG seropositive and seronegative patients	NMOSD attacks	Other neurological disease controls	Frequency and diagnostic value of CSF AQP4-IgG	AQP4-IgG (aquaporin-4)	NMOSD	CSF AQP4 is detectable during relapse; specific for NMOSD
Sato et al., 2014 [19]	Pilot NMOSD cohort during attacks	NMOSD attacks	NA/controls	CSF AQP4 levels and cytokine correlation	AQP4-IgG (quantified in CSF)	NMOSD	CSF AQP4 correlated with pleocytosis and IL-6, a marker of active disease
Caputi et al., 2022 [20]	2 RA patients with rheumatoid meningitis	RA with CNS involvement	NA (case-based)	CSF anti-CCP IgG and index; treatment response	Anti-CCP (anti-cyclic citrullinated peptide)	Rheumatoid arthritis - meningitis	Positive CSF anti-CCP index at diagnosis; decline after therapy

Risk-of-Bias Assessment

Table 2 presents the risk-of-bias assessment for the studies included in this systematic review. Fragoso-Loyo et al. conducted an observational cohort study of 47 patients with NPSLE and appropriate controls, with standardized CSF and serum autoantibody measurement and clear outcome reporting over a six-month follow-up, and were rated as low risk of bias using the NOS [11]. Similarly, Yoshio et al. investigated adults with NPSLE compared to non-NPSLE or neurological controls, using validated CSF anti-NR2 assays, and demonstrated low risk of bias [12]. Hirohata et al. and Arinuma et al. also conducted observational cohort studies with well-defined cohorts, clear control groups, validated assays, and clinical correlation, both rated low risk [13,14]. Hu et al. performed a pilot exploratory cohort study with a small sample size using proteome microarray techniques, and it was rated moderate risk due to its exploratory nature [15]. Duarte-García et al. conducted a prospective cohort study with an appropriate control group and objective cognitive outcomes, achieving a low risk rating [16]. Mégevand et al. performed a case series on CSF anti-SSA in pSS with CNS involvement, rated moderate risk due to limited generalizability [17]. Jarius et al. carried out a large observational cohort study on CSF AQP4 antibodies in NMOSD, rated low risk [18], while Sato et al. conducted a pilot cohort study on CSF AQP4 quantification and cytokine correlation, rated moderate risk [19]. Finally, Caputi et al. described a case series of two patients with RA and rheumatoid meningitis, including CSF anti-CCP measurements, which was rated as moderate risk due to the very small sample size [20]. This assessment highlights the methodological quality and potential limitations of included studies, providing context for interpreting the reported CSF autoantibody findings. 

**Table 2:** Risk-of-bias assessment. CSF, cerebrospinal fluid; NPSLE, neuropsychiatric systemic lupus erythematosus; SLE, systemic lupus erythematosus; pSS, primary Sjögren’s syndrome; NMOSD, neuromyelitis optica spectrum disorder; RA, rheumatoid arthritis; NOS, Newcastle-Ottawa Scale; JBI, Joanna Briggs Institute; AQP4, aquaporin-4; CCP, cyclic citrullinated peptide

Study	Study design	Risk-of-bias tool	Risk-of-bias rating	Justification
Fragoso-Loyo et al., 2008 [11]	Observational cohort	NOS	Low	A well-defined cohort of 47 patients with NPSLE, with appropriate controls; standardized CSF and serum autoantibody measurements; and clear outcome reporting with a six-month follow-up.
Yoshio et al., 2006 [12]	Observational cohort	NOS	Low	Adults with NPSLE compared to non-NPSLE or neurological controls; validated CSF anti-NR2 assays; clearly described selection and outcome assessment; and outcomes correlated with neuropsychiatric manifestations.
Hirohata et al., 2007 [13]	Observational cohort	NOS	Low	A well-defined cohort, a clear control group, a standardized anti-P assay, and clinical correlation were included.
Arinuma et al., 2008 [14]	Observational cohort	NOS	Low	Adequate selection and outcome assessment, CSF anti-NR2 measured with a validated method.
Hu et al., 2015 [15]	Pilot cohort / exploratory	NOS	Moderate	Small sample size, exploratory proteome array, but methodology clearly described.
Duarte-García et al., 2018 [16]	Prospective cohort	NOS	Low	Proper control group, outcome (cognitive function) objectively measured, CSF biomarkers clearly described.
Mégevand et al., 2007 [17]	Case series	JBI Checklist	Moderate	Small sample, CSF anti-SSA measured accurately, but limited generalizability.
Jarius et al., 2010 [18]	Observational cohort	NOS	Low	Large cohort, standardized CSF AQP4 antibody measurement, and clear diagnostic criteria.
Sato et al., 2014 [19]	Pilot cohort	NOS	Moderate	Small sample, but rigorous CSF AQP4 quantification and cytokine correlation.
Caputi et al., 2022 [20]	Case series	JBI Checklist	Moderate	Very small sample (*n* = 2), well-described CSF anti-CCP assay, useful for diagnostic insight but limited external validity.

Discussion

This systematic review highlights the clinical and diagnostic significance of CSF autoantibodies in rheumatologic disorders affecting the CNS. CSF analysis provides a unique window into intrathecal immune activity, which is often more specific to CNS pathology than serum measurements alone. In NPSLE, anti-NR2 (N-methyl-D-aspartate receptor subunit 2) and anti-ribosomal P antibodies are consistently associated with cognitive dysfunction, mood disorders, and diffuse psychiatric manifestations, emphasizing their potential role not only as diagnostic biomarkers but also as indicators of disease activity and severity [11-15]. Detection of these antibodies in CSF, rather than serum, enhances specificity by reflecting direct CNS immune involvement and may guide timely therapeutic interventions, particularly immunosuppressive strategies, before irreversible neuronal damage occurs.

In pSS with CNS involvement, intrathecal synthesis of anti-SSA (Ro) antibodies has been demonstrated, suggesting localized autoimmune activation in the CNS [17]. These findings underscore the importance of CSF analysis in differentiating CNS manifestations of systemic autoimmune diseases from other neurological disorders, such as multiple sclerosis or infectious encephalopathies. Similarly, in NMOSD, CSF aquaporin-4 immunoglobulin G (AQP4-IgG) correlates with active relapses, pleocytosis, and proinflammatory cytokines like IL-6, highlighting both diagnostic and prognostic utility [18,19]. Such insights can inform personalized treatment strategies, including early initiation of targeted immunotherapy to prevent relapse and permanent neurological deficits.

In RA with CNS involvement, although rare, CSF anti-cyclic citrullinated peptide (anti-CCP) antibodies have been detected in cases of rheumatoid meningitis, serving as a specific biomarker for CNS-directed autoimmune inflammation [20]. These observations expand the spectrum of CSF autoantibody testing beyond classical SLE and NMOSD, emphasizing the broader relevance of intrathecal antibody profiling in rheumatologic CNS disease.

However, several limitations exist. Most studies were observational, often with small sample sizes and heterogeneous methodology in CSF collection, antibody quantification, and outcome measurement. Longitudinal correlations between CSF autoantibody titers and clinical disease progression or therapeutic response are limited, restricting our ability to establish predictive thresholds for relapse or treatment efficacy. Future research should focus on multicenter, prospective cohorts with standardized CSF sampling protocols and validated assays, incorporating longitudinal follow-up and integration with neuroimaging, clinical scales, and serum biomarkers. Advancing such studies may allow the development of precise biomarker panels that can guide early diagnosis, prognostication, and personalized immunotherapy in patients with rheumatologic CNS involvement.

## Conclusions

CSF autoantibody profiling provides valuable insights into CNS involvement in rheumatologic disorders, including SLE, pSS, NMOSD, and RA. Disease-specific antibodies, such as anti-NR2, anti-ribosomal P, anti-SSA (Ro), AQP4-IgG, and anti-CCP, not only aid in accurate diagnosis but also correlate with neurological and psychiatric manifestations, disease activity, and prognosis. Integrating CSF autoantibody analysis into clinical practice can enhance diagnostic specificity, guide timely therapeutic interventions, and support personalized patient management. Future studies with standardized methodologies and longitudinal follow-up are essential to establish predictive biomarker panels, optimize treatment strategies, and improve outcomes in patients with rheumatologic CNS involvement.
